# Assessment of COVID-19 Incidence and the Ability to Synthesise Anti-SARS-CoV-2 Antibodies of Paediatric Patients with Primary Immunodeficiency

**DOI:** 10.3390/jcm10215111

**Published:** 2021-10-30

**Authors:** Karolina Pieniawska-Śmiech, Anna Kuraszewicz, Joanna Sado, Karol Śmiech, Aleksandra Lewandowicz-Uszyńska

**Affiliations:** 1 Department of Clinical Immunology, Wroclaw Medical University, 50-368 Wroclaw, Poland; 2 Department of Clinical Immunology and Paediatrics, Provincial Hospital J. Gromkowski, 51-149 Wroclaw, Poland; akuraszewicz@gmail.com (A.K.); asia.sado@gmail.com (J.S.); 3 Department of Cardiology, Research and Development Center, Regional Specialist Hospital, 51-124 Wroclaw, Poland; smiech.karol@gmail.com; 4 3rd Department and Clinic of Paediatrics, Immunology and Rheumatology of Developmental Age, Wroclaw Medical University, 50-367 Wroclaw, Poland

**Keywords:** anti-SARS-CoV-2 antibodies, COVID-19, primary immunodeficiency, SARS-CoV-2, PIMS-TS

## Abstract

Background: Data regarding the course of SARS-CoV-2 infection in children with primary immunodeficiency (PID) is insufficient. The purpose of the study was to evaluate the morbidity and clinical course of COVID-19 and the ability to produce anti-SARS-CoV-2 IgG antibodies in children with PID. Methods: In this retrospective study, medical records of 99 patients aged 0–18 were evaluated. The patients were divided into three groups: PID group (68.69%), control group (19.19%) and patients with ongoing or previous paediatric inflammatory multisystem syndrome (12.12%). Data such as morbidity, clinical outcome, and IgG anti-SARS-CoV-2 antibody titres were assessed. Results: A confirmed diagnosis of SARS-CoV-2 infection has been established in 26.47% of patients with PID. Among patients with PID infected with SARS-CoV-2, only three cases were hospitalised. Mortality in the PID group was 0%. Throughout an observation period of 1 year, 47.06% of patients with PID were tested positive for the anti-SARS-CoV-2 antibody. Conclusions: In the study group, in most cases the disease had a mild and self-limiting course. Remarkably, even though IgG deficiency was the most prevalent form of PID in the study group, the patients were able to respond satisfactorily to the infection in terms of anti-SARS-CoV-2 IgG.

## 1. Introduction

At the end of 2019, a new strain of pneumonia-causing coronavirus was identified in Wuhan, China [1]. Its rapid spread resulted in an outbreak of an epidemic that started in China and gradually expanded worldwide [2,3]. The causative virus, initially called 2019-nCoV, was named SARS-CoV-2, and the disease associated with it–COVID-19 [4].

Primary immunodeficiency (PID) manifests mainly as recurrent and/or severe infections, and patients affected by PID constitute a unique population [5]. If possible, medical interventions should focus on correcting the immune defect in the first place. Another important goal is the prevention and treatment of infections that are still an important cause of mortality in this patient group.

The Center for Disease Control and Prevention (CDC) recognises PID as a risk factor of severe clinical course of COVID-19 [6,7]. Data regarding the course of SARS-CoV-2 infection in children, including children with PID, are insufficient. Other areas that need further research are the duration time of immunity to reinfection and the applicability of serological methods in confirming previous infections [8,9,10,11,12]. Following appropriate validation, serologic tests detecting anti-SARS-CoV-2 antibodies might help identify patients who were infected with the new coronavirus in the past [13,14,15,16]. To increase the predictive value of serological methods, it has been suggested that only the tests with high specificity (>99.5%) were used only on individuals with a high clinical probability of a previous infection. The main disadvantages of serologic tests are limited use in the diagnosis of the acute phase of the infection, variable sensitivity and specificity, depending on the assay, and relatively high costs and absence of antibody synthesis in response to the infection in some patients [17]. It is obvious that such a phenomenon may be observed in patients with immune deficiency, both congenital and acquired. The purpose of the study was to evaluate the morbidity and clinical course of COVID-19 and the ability to produce the anti-SARS-CoV-2 IgG antibodies in children with PID. At the same time, the applicability of serological methods in the diagnosis of SARS-CoV-2 in this group of patients was assessed.

## 2. Materials and Methods

In this retrospective study medical records of 99 patients aged 0–18 who were admitted to the Department of Clinical Immunology and Paediatrics of J. Gromkowski Provincial Hospital in Wrocław from June 2020 to June 2021 were assessed. Testing for coronavirus infection (antigen/polymerase chain reaction-PCR tests) and serologic tests for IgM and IgG anti-SARS-CoV-2 antibodies titres were performed on all of the included patients. Samples were collected from June 2020 to June 2021, during hospitalisation. The patients were divided into three groups: patients already diagnosed with PID according to IUIS criteria and classification (study group) accounted for 68.69% (*n* = 68), patients without an established diagnosis of PID (control group) constituted 19.19% (*n* = 19), and patients with ongoing or previous paediatric inflammatory multisystem syndrome (PIMS-TS/MIS-C) constituted 12.12% (*n* = 12). The control group consisted of patients with recurrent respiratory tract infections diagnosed in the Department of Immunology and Paediatrics who did not show abnormalities in immunological tests and did not meet IUIS criteria for inborn errors of immunity (IEI).

A total of 63.64% (*n* = 63) of the patients were male, and 36.36% (*n* = 36) were female. The mean age of the patients was 7.3 years. All the patients in the study group had been managed in the department for their PID before—55.88% (*n* = 38) of them were treated with immunoglobulin substitution therapy, and the remaining 44.12% (*n* = 30) did not receive such treatment. The most common form of PID was antibody deficiency (*n* = 44) (Table 1).

IgM and IgG anti-SARS-CoV-2 antibody titres were measured quantitatively using chemiluminescence. All of the tests were performed in the same laboratory. IgG antibodies against S1/S2 antigens of SARS-CoV-2 were measured from June 2020 to March 2021 and the anti-trimeric spike glycoprotein of SARS-CoV-2 IgG antibodies were measured from March 2021 to June 2021.

Statistical analysis of data was conducted using the spreadsheet of Microsoft Office Excel (Microsoft Corp., Redmond, WA, USA) and Statistica v. 13–non-parametric Mann-Whitney U test. The significance level was defined as α = 0.05. A *p*-value less than 0.05 was considered statistically significant.

Consent for the study was granted by the Bioethics Committee of the Wroclaw Medical University.

## 3. Results

### 3.1. Morbidity and Disease Course

**Figure 1:**
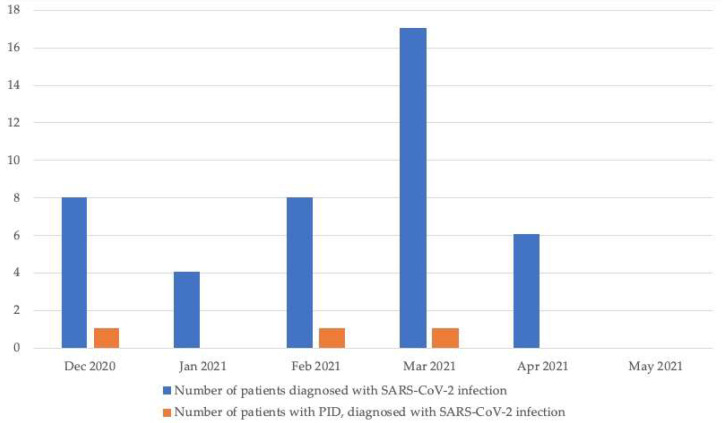
Number of cases of acute SARS-CoV-2 infection identified as a result of the routine testing performed before hospital admission.

Throughout an observation period of 1 year, a diagnosis of SARS-CoV-2 infection was confirmed (by means of a PCR or antigen laboratory test) in 18 out of 68 patients with PID (26.47%). Three cases were diagnosed incidentally during tests before non-COVID-19-related hospital admission (Figure 1). Signs of COVID-19 and/or high probability of the infection (e.g., positive result of a SARS-CoV-2 test in a close family member) were identified in 13 patients (19.12%).

Most of the confirmed and/or highly probable cases of COVID-19 (*n* = 19; 61.29%) were noted during the so-called ‘second wave’ of the pandemic (September 2020–January 2021, and none during ‘first wave’ (March 2020–August 2020). The predominant variant of SARS-CoV-2 in Poland at the end of 2020 and the beginning of 2021 was 20A but when 20I (also known as B.1.1.7) variant emerged in late December 2020, it quickly became the one responsible for the largest number of infections and started the so-called ‘third wave’.

The most common symptom of the infection was elevated body temperature: fever (*n* = 12) or low-grade fever (subfebrile temperature; *n* = 6) (Table S1). Among patients with PID infected with SARS-CoV-2, hospital admission was necessary in only three cases (4.41%)—each of those children suffered from humoral immune disorders, one of them also had a diagnosis of Rubinstein-Taybi syndrome. One of the hospitalised patients required oxygen therapy and was treated with convalescent plasma, none of them required management in an intensive care unit. As for June 2021, mortality in the PID group was 0%.

### 3.2. Evaluation of Anti-SARS-CoV-2 Antibody Synthesis

Throughout an observation period of 1 year, 32 out of 68 patients with PID tested positive for anti-SARS-CoV2 antibodies (47.06%). By far the majority of these cases (*n* = 31; 96.88%) were associated with confirmed or highly probable (close contact with an infected individual, e.g., a parent, and/or symptoms characteristic of COVID-19) SARS-CoV-2 infection. Furthermore, among 18 patients with a positive test result for SARS-CoV-2 genetic material, only 2 (11.11%) did not produce antibodies directed against it—this included a 12 month-old child with IgG and IgA deficiency and a patient with Rubinstein-Taybi syndrome and IgA, IgM and IgG deficiency, who was also treated with convalescent plasma during the acute phase of the disease.

Among patients with PID who were infected or were most likely infected with COVID-19, there were 10 children (14.70% of all patients with PID) who have been treated with immunoglobulin substitution therapy at that time and only one of these patients required hospital admission. It is also noteworthy that a patient with a history of severe combined immune deficiency (SCID) and hematopoietic stem cell transplantation, which was performed a few years before, developed anti-SARS-CoV-2 antibodies following a symptomatic infection with the virus. Moreover, there was no need for hospital admission in this case.

**Figure 2:**
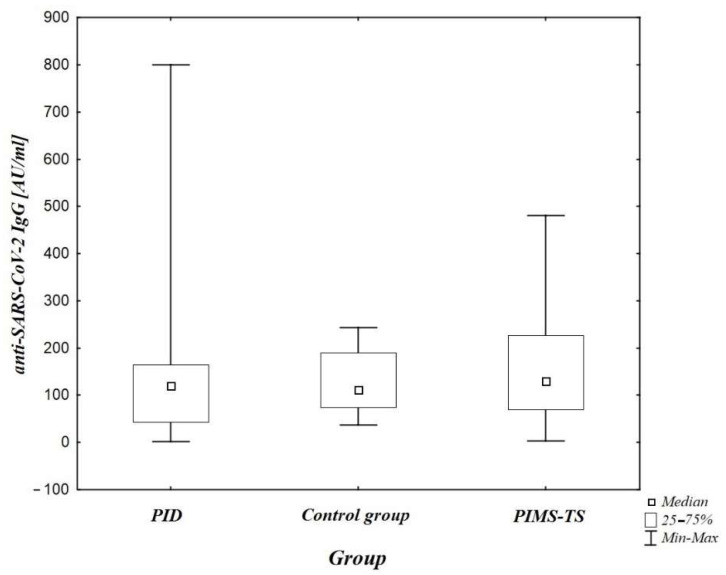
A comparison of anti-SARS-CoV-2 IgG antibody levels in patients with confirmed or highly probable COVID-19 in each patient group (*p* > 0.05). PID-primary immunodeficiency; PIMS-TS-paediatric inflammatory multisystem syndrome.

As for the levels of IgG anti-SARS-CoV-2 antibodies in individuals with positive test results, there were no statistically significant differences when compared with the control group (*n* = 19) (*p* > 0.05) (Figure 2), as well as between the PID group and patients with ongoing or previous PIMS-TS and between the control group and the PIMS-TS group.

**Figure 3:**
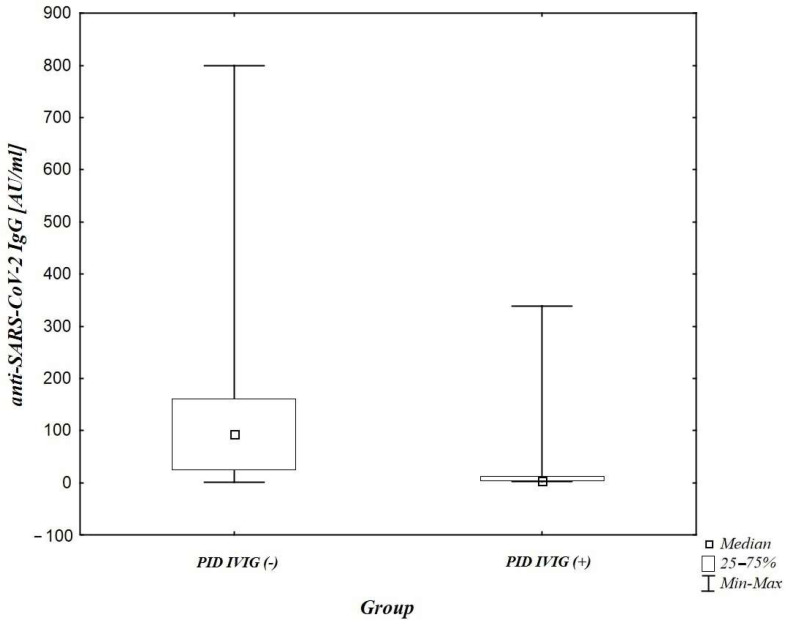
A comparison of anti-SARS-CoV2 IgG antibody levels between patients with PID receiving immunoglobulin substitution therapy (PID IVIG (+)) and patients with PID without such treatment (PID IVIG (−)) (*p* = 0.0001).

However, a statistically significant difference (*p* = 0.0001) between patients with PID receiving immunoglobulin substitution therapy and patients with PID without such treatment was noted (Figure 3).

## 4. Discussion

In the absence of more extensive and/or thorough data, it remains unclear whether PID is a predisposing or, paradoxically, a protective factor for SARS-CoV-2 infection [18]. To answer this question, more data regarding COVID-19 morbidity, clinical course and mortality in patients with PID is necessary. Our study represents the experiences of one clinical centre and as such should be regarded as a single opinion in a broader discussion. It is worth considering if immunoglobulin replacement therapy is the protective factor for a severe course of COVID-19, even if immunoglobulins available on the market during the study (June 2020–June 2021) probably did not contain significant level of anti-SARS-CoV-2 IgG antibodies. This influence may be associated with the modulatory effect of immunoglobulins on the immune system, which is used in therapy of e.g., Kawasaki disease, Guillain-Barre syndrome [19].

In an observational study conducted in Israel [20], which was published in January 2021, amongst patients with PID aged 4 months to 6 years, a total number of 20 SARS-CoV-2 infections was recorded. The majority of these cases (95%) were reported during the second wave of the pandemic, which was consistent with our results. Moreover, children receiving immunoglobulin substitution therapy constituted the majority of the infected patient population. There were no cases of severe COVID-19, none of the infected patients required hospital admission and 35% of the affected children remained asymptomatic during the course of the disease. The authors implied that the COVID-19 pandemic had little impact on patients with PID.

The observations made by researchers in Iran [21] were different. In a prospective study, based on data acquired from the national registry, it was concluded that with only 1.23-fold higher incidence of infections, patients with PID, mainly those with combined immunodeficiency and immune dysregulation, present a 10-fold higher mortality rate compared to the general population. The study included 19 children with PID in whom SARS-CoV-2 infection was confirmed using an RT-PCR test. Exposure to the virus from an unknown source or a source outside the patient’s family accounted for 84.2% of the total number of cases. The results of our study were quite different, and contact with close relatives was the source of infection for many of the infected patients (*n* = 11).

The Iranian researchers showed that combined immunodeficiency (*n* = 10, all without hematopoietic stem cell transplantation or HSCT, 47.0%) was the major PID entity amongst COVID-19 positive cases, followed by humoral immunodeficiencies (*n* = 4), phagocytic defects (*n* = 2), immune dysregulation (*n* = 2), and autoinflammatory disorders (*n* = 1) [21], ergo studied population was different than ours. The discrepancies in the incidence of certain forms of PID might be a result of the relatively low number of patients in both study groups and different characteristics of the populations managed in each hospital department. It is worth mentioning that the difference between Iranian and Polish patients with PID is related to the high prevalence of consanguinity in Iran compared to Poland, and the consequent high prevalence of autosomal recessive immunodeficiency.

In an international study conducted by Meyts et al. [22] published in February 2021, 32 cases of COVID-19 were recorded in children with PID, nine of them required management in an intensive care unit (ICU) and two of them died. Among patients treated in the ICU settings there were patients with a diagnosis of chronic granulomatous disease (*n* = 1), trisomy 21 (*n* = 1), Wiskott-Aldrich syndrome (*n* = 1), nuclear factor κB mutation (NFKB2) (*n* = 1) and X-linked inhibitor of apoptosis protein (XIAP) deficiency (*n* = 1). Due to numerous comorbidities, the authors defined the connection between SARS-CoV-2 infection and the death of both patients as ‘unclear’.

Throughout an observation period of 1 year, none of the patients managed by our department for PID required treatment in ICU while infected with SARS-CoV-2.

At present, the data regarding IgG anti-SARS-CoV-2 antibody synthesis in individuals with PID are insufficient.

However, the research conducted on the immunocompetent population presents some interesting information. A key factor in determining the appropriate time window for the use of serological tests is the occurrence of seroconversion. Recent publications indicate that the median IgG detection occurs 9 to 14 days after disease onset [23,24]. Peterson et al. reported that approximately 1 in 16 people lacked IgG antibodies following infection. Race/ethnicity, weight status, immunosuppressive therapy and illness severity were independent predictors of IgG antibody presence after SARS-CoV-2 infection [25].

In a study conducted by Venkatamaran et al. in India, the authors evaluated humoral immune response associated with anti-SARS-CoV-2 antibody synthesis in hospitalised patients by comparing antibody titres between children with and without PIMS-TS [26]. Almost half of seropositive children had PIMS-TS. Antibody levels may be helpful in the diagnosis and disease stratification of PIMS-TS. Nearly one-fifth of the hospitalised children tested serology positive over four months. Antibody levels in children with PIMS-TS were significantly higher in comparison to the other two groups (acute COVID-19 infection and children without PIMS-TS).

The main purpose of our study was to evaluate antibody synthesis in patients with primary immune deficiency, and so an additional comparison of antibody synthesis between this unique population and children with (both ongoing and previous) PIMS-TS was made. No difference in levels of the antibody was recorded (Figure 2). This observation requires further verification on a larger group of patients, including meticulous evaluation of synthesis and perseverance of the antibody during the acute phase of the disease and after its resolution. It is noteworthy that in one patient with a history of PIMS-TS, IgG subclass deficiency was detected twice. There was no reference, however, since no immunological studies were performed in this patient before PIMS occurred.

It is also important to note that amongst children receiving immunoglobulin substitution therapy, positive anti-SARS-CoV-2 IgG test was recorded only in individuals with infection confirmed with a PCR/antigen test or with a high probability of infection, which meant the presence of characteristic clinical symptoms and/or close contact with an infected person. Moreover, the mean level of anti-SARS-CoV-2 IgG in this group (IVIG +) was lower than in children not receiving such treatment and proved to be statistically significant (*p* = 0.0001). This leads to the conclusion that the IgG anti-CoV antibody test results were legitimate (throughout the observation period). Another important issue is that a significant percent of the study group with relatively mild immunodefciency (= not required immunoglobulin replacement therapy at the time of the study, i.e., isolated IgG subclass deficiency) may influence the results of the study.

Furthermore, it is worth mentioning, that the control group was not a classic control group and included patients with (mostly) mild recurrent upper respiratory tract infections, without abnormalities in immunological tests, who did not meet IUIS criteria for IEI.

The most important limitation of the study was the retrospective nature of the analysis which was based on collected medical records. More precise, prospective studies, evaluating the duration of antibody response in patients with PID and a history of COVID-19 are needed.

## 5. Conclusions

COVID-19 might be regarded as one of the main challenges for healthcare in the twenty-first century. However, based on the ongoing collection of data, it will be possible to identify the risk group of severe COVID-19 amongst individuals with PID in the future. Patients diagnosed with PID constitute a unique population. Usually, they are provided with high-quality medical care and were well isolated throughout the pandemic. Moreover, the caregivers responsible for them are fully aware of the danger and abide by all of the hygiene standards. As a result, throughout the first wave of the pandemic, the number of infections detected in patients with PID was much smaller than in immunocompetent patients. The second and the third wave were associated with an increase in the number of infections both in adults and in children. Many of the patients managed by the department for their PID became infected at that time. However, in most cases, the disease had a mild and self-limiting course. Study results indicate that COVID-19 is not only a less severe disease in children than in adults, but also is not as severe as one might expect in children with dysfunctional immune systems. Nevertheless, this observation should not affect the sanitary regime and safety regulations concerning the management of PID patients, especially in the context of the new B.1.617.2 (delta) variant.

Remarkably, even though the most prevalent form of PID in the study group was IgG deficiency, the patients were able to respond satisfactorily to the infection in terms of anti-SARS-CoV-2 IgG. Thus, some of PID may be a group with a significance in limitation of transmission of SARS-CoV-2 viral infection after COVID-19 vaccination. According to Polish consensus by group of experts, vaccination against COVID-19 should be recommended [27].

It seems that the main factor influencing the course of COVID-19 in both immunocompetent patients and the patients with PID is comorbidity.

## Figures and Tables

**Table 1:** Types of PID and its prevalence in the study group.

Primary Immunodeficiency	Number of Patients; Percent
Combined immunodeficiencies with associated or syndromic features	*n* = 20; 29.14%
IgG subclass deficiency	*n* = 18; 26.47%
Hypogammaglobulinemia IgG	*n* = 14; 20.59%
Other hypogammaglobulinemias *	*n* = 8; 11.76%
Common variable immunodeficiency (CVID)	*n* = 3; 4.11%
Severe combined immunodeficiency (SCID)	*n* = 2; 2.94%
Other, unclassified **	*n* = 2; 2.94%
X-linked agammaglobulinemia	*n* = 1; 1.01%

Abbreviations: * IgG subclass deficiency with IgA deficiency/selective IgM deficiency/transient hypogammaglobulinemia of infancy/IgM and IgG subclass deficiency; ** isolated congenital asplenia/severe lymphocyte T deficiency during diagnostics.

## Data Availability

The authors confirm that the data supporting the findings of this study are available within the article and its supplementary materials.

## Supplementary Materials

The following are available online at www.mdpi.com/article/10.3390/jcm10215111/s1, Table S1: Clinical characteristics of patients with PID with confirmed or suspected SARS-CoV-2 infection.
