# Supplementary material for: Assessment of COVID-19 Incidence and the Ability to Synthesise Anti-SARS-CoV-2 Antibodies of Paediatric Patients with Primary Immunodeficiency
Source: J Clin Med. 2021 Oct 30;10(21):5111. doi: 10.3390/jcm10215111 (PMC8584568; doi:10.3390/jcm10215111)
Supplement: Supplementary file 1 [file jcm-10-05111-s001.zip › jcm-1428164-supplementary.pdf]

Journal of
Clinical Medicine

MDPI

Supplementary Materials

Table S1. Clinical characteristics of patients with PID with confirmed or suspected SARS-CoV-2 infection.

Patient No	Sex	Age (years)	PID diagnosis	COVID-19 infection	Symptoms of COVID-19	Antibody anti-SARS-CoV-2 production [AU/ml]	IgG [mg/dL] [norm]	Outcome
**1**	M	2,5	IgG deficiency	Yes	Fever, cough, diarrhoea	+, 169	454,0 [540-1822]	Recovery
**2**	F	12	IgG deficiency	Yes	Fever, sore throat	+, 113	410,1 [553-1232]	Recovery
**3**	M	1,5	DiGeorge syndrome	Yes	Anxiety, bradycardia	+, 27,7	189,8 [119-790]	Recovery
**4**	F	9	Silver-Russel syndrome	Probably	Fever, sore throat, cough, headache	+, 77,8	425,0 [553-1232]	Recovery
**5**	M	9	Nijmegen breakage syndrome	Yes	Upper respiratory tract infection, fever, rhinorrhoea	+, 26,1	827 [540-1822]	Recovery
**6**	M	8	IgG subclass deficiency	Probably	Fever, myalgia, rhinorrhoea	+, 247	1143,0 [553-1232]	Recovery
**7**	F	4	Kabuki syndrome, IgG deficiency	Yes	Fever, diarrhoea, rhinorrhoea, earache	+, 93	403,0 [553-1631]	Recovery
**8**	M	7	IgG subclass deficiency	Probably	Subfebrile state, cough	+, 85	1070,0 [540-1822]	Recovery
**9**	F	7,5	IgG subclass deficiency	Probably	Subfebrile state, rhinorrhoea	+, 25	751,0 [553-1631]	Recovery
**10***	M	6	SCID (IL2RG)	Probably	Fever, cough	+, 338	649,1 [297-969]	Recovery
**11**	F	7	IgM, IgG subclass deficiency	Probably	Asymptomatic	+, 50	1050,0 [553-1631]	Recovery

**12**	M	6	IgG deficiency	Yes	Upper respiratory tract infection, elevated CRP 80 mg/l	+, 120	356,6 [408-1120]	Recovery
**13**	F	3,5	Nijmegen breakage syndrome	Probably	Rhinorrhoea	+, 25,7	117,2 [119-790]	Recovery
**14**	M	9	IgG subclass deficiency	Probably	Fever for 5 days	+, 38	500,3 [468-1150]	Recovery
**15**	F	1,5	IgG deficiency	Probably	Diarrhoea	+, 148	457,0 [483-1226]	Recovery
**16**	M	12,5	IgG subclass deficiency	Yes	Mild infection symptoms, rhinorrhoea, cough	+, 144	1300,0 [518-1284]	Recovery
**17**	M	16,5	Rubinstein-Taybi syndrome, IgM, IgG, IgA deficiency	Yes	Subfebrile state, rhinorrhoea, cough, thrombocytopenia, desaturation	-, 5,96	<108,5 [518-1284]	Hospitalization Required oxygen administration Recovery
**18**	F	4,5	IgM deficiency	Probably	Lack of data	+, 32,7	778,0 [553-1631]	-
**19**	M	3,5	IgG subclass deficiency	Yes	Asymptomatic	+, 219	456,0 [408-1120]	-
**20**	F	1,5	IgG deficiency	Yes	Fever, encephalitis suspicion	+, 447	448,0 [483-1126]	Hospitalization, Recovery
**21**	M	8,5	IgG subclass deficiency, trisomy 21	Probably	Subfebrile state	+, 65,6	485,2 [297-969]	Recovery
**22**	F	12,5	CD3+, CD4+, CD8+ deficiency during diagnostics	Yes	Mild symptoms, cough	+, 192	1111,0 [553-1631]	Recovery
**23**	F	1	IgA, IgM, IgG	Yes	Subfebrile state	+, >800	461,4 [483-1226]	Recovery

			deficiency					
**24**	M	4,5	IgG subclass deficiency	Yes	Mild symptoms	+, 151	655,0 [540-1882]	Recovery
**25**	F	1	IgG, IgA deficiency	Yes	Fever, cough	-, <1,85	440,0 [483-1226]	Hospitalized, Recovery
**26**	M	11	IgG subclass deficiency	Probably	Asymptomatic /mild symptoms	+, 42,5	819,0 [540-1823]	
**27**	M	11	Ataxia-telangiectasia	Probably	Fever, rhinorrhoea,cough	+, 47,2	802,0 [540-1822]	Recovery
**28**	M	9,5	IgG subclass deficiency	Yes	Cough, dyspnea, pneumonia	+, 136	668,0 [540-1822]	Recovery
**29**	M	2	IgG subclass deficiency	Probably	Fever	+, 139	380,0 [349-918]	Recovery
**30**	M	7	IgG subclass deficiency	Yes	Sore throat	+, 136	775,0 [540-1822]	Recovery
**31**	M	3	IgG deficiency	Yes	Cough, asthma exacerbation	+, 213	536,0 [540-1822]	Recovery
**32**	M	6,5	Nijmegen breakage syndrome	Probably	Diarrhoea	+,29,5	301,1 [408-1120]	Recovery
**33**	F	8	Congenital asplenia, lymphocyte deficiency	Yes	Mild symptoms, subfebrile state, diarrhoea	+, 161	1296,1 [553-1631]	Recovery
**34**	M	4,5	IgG subclass deficiency	Probably	Asymptomatic when close contact to infected parent; then bronchitis without SARS-CoV-2 test	+, 164	608,0 [553-1631]	Recovery
**35***	F	15,5	IgG subclass	Yes	Fever, pneumonia	+, 84,9	1187,0 [553-1631]	MIS-C Recovery

	deficienc y

10* - patient with SCID after HSCT few years ago; data about immunoglobulin G level before substitution currently unavailable; 35* - patient assigned to MIS-C/ PIMS-TS group

Abbreviations: COVID-19 – coronavirus disease; CVID – common variable immunodeficiency; F- female; IgA – immunoglobulin A; IgG – immunoglobulin G; IgM – immunoglobulin M; M - male; MIS-C – multisystem inflammatory syndrome in children; No – number; PID – primary immunodeficiency; SARS-CoV-2 – severe acute respiratory syndrome coronavirus 2; SCID – severe combined immunodeficiency; + means anti-SARS-CoV-2 IgG >= 13 AU/ml and was considered positive result; - means anti-SARS-CoV-2 IgG <13 and was considered negative result
